# Association Between Metformin Use and Risk of Lung Cancer in Patients With Type 2 Diabetes: A Systematic Review and Meta‐Analysis

**DOI:** 10.1002/cnr2.70651

**Published:** 2026-08-11

**Authors:** Mehdi Karimi, Farnaz Hooshmand, Kiana Mohammadian, Seyed Morteza Ali Pourfaraji, Masoud Mortezazadeh

**Affiliations:** ^1^ Faculty of Medicine Bogomolets National Medical University (NMU) Kyiv Ukraine; ^2^ Department of Hematology‐Oncology, Cancer Institute, Imam Khomeini Hospital Complex Tehran University of Medical Sciences Tehran Iran; ^3^ Faculty of Medicine Golestan University of Medical Sciences Gorgan Iran; ^4^ Division of Hematology and Blood Banking, Department of Medical Laboratory Sciences, School of Paramedical Sciences Shiraz University of Medical Sciences Shiraz Iran; ^5^ Faculty of Medicine Tehran University of Medical Sciences Tehran Iran

**Keywords:** diabetes, epidemiology, lung Cancer, meta‐analysis, metformin, risk

## Abstract

**Background and Aim:**

Lung cancer remains one of the leading causes of cancer‐related mortality worldwide. Type 2 diabetes mellitus (T2DM), the most prevalent endocrine disorder, is commonly managed with Metformin, an antidiabetic medication that has demonstrated potential anticancer properties. This meta‐analysis aimed to discover the association between Metformin use and the risk of lung cancer in patients with T2DM.

**Methods:**

A comprehensive search of online databases was conducted up to January 1, 2026, to identify relevant observational studies. After screening, appropriate data were extracted. Meta‐analyses utilized the most fully adjusted hazard ratio (HR) with corresponding 95% confidence intervals (CI) as the effect measures. All statistical analyses were performed using R Statistical Software.

**Results:**

The pooled analysis of 29 observational studies showed a significant reduction in lung cancer risk among Metformin users compared with nonusers (HR: 0.85, 95% CI: [0.77–0.94], *p* = 0.002). Subgroup analyses revealed significant associations in cohort studies (HR: 0.79, 95% CI: [0.70–0.89], *p* < 0.01) and in studies conducted in Asia (HR: 0.82, 95% CI: [0.68–0.99], *p* = 0.04). Extended Metformin use (> 3 years) was associated with a significant reduction in lung cancer risk (HR: 0.87, 95% CI: [0.79–0.95]; *p* < 0.01), whereas no significant effect was observed for shorter durations.

**Conclusion:**

This meta‐analysis indicated a potential protective effect of Metformin use in individuals with T2DM against lung cancer, with a significant risk reduction observed, particularly among long‐term users and in Asian populations. The significant associations identified in cohort studies and in extended‐duration use underscore metformin's promise in lung cancer prevention.

**Abbreviations**
CI: confidence interval
HR: hazard ratios
OR: odds ratio
RR: relative risks
T2DM: Type 2 diabetes mellitus

## 1 Introduction

Lung cancer remains the leading cause of cancer‐related mortality worldwide, accounting for more than 2 million new cases annually [1]. Despite advances in diagnosis and treatment, the prognosis of lung cancer remains poor, particularly in advanced‐stage disease, highlighting the need for effective preventive and therapeutic strategies [2]. Type 2 diabetes mellitus (T2DM), a rapidly growing global health burden, has been associated with an increased risk of both lung cancer incidence and lung cancer–specific mortality [3, 4]. Several biological mechanisms, including chronic hyperglycemia, insulin resistance, hyperinsulinemia, and activation of insulin‐like growth factor (IGF) signaling pathways, have been proposed to promote tumor initiation and progression in individuals with T2DM [5]. Given the widespread use of metformin as a first‐line therapy for T2DM, considerable interest has emerged regarding its potential anticancer properties. Experimental and epidemiological studies suggest that metformin may inhibit cancer development and progression through multiple mechanisms, including modulation of cellular metabolism, suppression of proliferative signaling pathways, and reduction of systemic insulin levels [6, 7]. These observations underscore the importance of investigating the relationship between metformin use and lung cancer risk and outcomes among patients with T2DM.

Metformin, a widely prescribed first‐line therapy for T2DM, has attracted considerable attention for its potential anticancer properties. Over the past decade, numerous epidemiological, clinical, and experimental studies have investigated the association between metformin use and the risk, progression, and prognosis of various malignancies, including gastric cancer [8], bladder cancer [9], hepatocellular carcinoma [10], colorectal cancer, breast cancer [11], endometrial cancer [12], and thyroid cancer [13]. Proposed mechanisms underlying these effects include activation of AMP‐activated protein kinase (AMPK) [14], inhibition of the mammalian target of rapamycin (mTOR) signaling pathway [15], reduction of insulin and insulin‐like growth factor‐1 (IGF‐1) signaling, and suppression of tumor cell proliferation and metastasis [16]. Despite the substantial body of evidence regarding metformin and cancer, findings remain inconsistent across cancer types and populations, highlighting the need for further investigation into its potential role in cancer prevention and treatment.

Despite encouraging evidence from experimental studies and epidemiological investigations, the association between metformin use and lung cancer risk remains uncertain. Several observational studies have reported a potential protective effect of metformin against lung cancer development [17, 18, 19], whereas others have observed no significant association [20, 21, 22]. Moreover, previous studies have suggested lower cancer incidence and reduced lung cancer–related mortality among patients with T2DM receiving metformin compared with nonusers [23, 24]. These inconsistent findings may be attributable to differences in study design, population characteristics, exposure assessment, adjustment for confounding factors, and geographic settings. Although metformin has attracted considerable interest as a potential anticancer agent, the available evidence regarding its role in lung cancer prevention remains fragmented and has not been comprehensively synthesized.

Therefore, a systematic review and meta‐analysis is warranted to provide a more precise estimate of the association between metformin use and lung cancer risk. The present study aims to systematically evaluate and quantitatively synthesize evidence from observational studies examining this relationship among patients with T2DM. In addition to generating a pooled risk estimate, this study explores potential sources of heterogeneity through subgroup and sensitivity analyses. By integrating the currently available evidence, this review offers updated, comprehensive insights into the potential role of metformin in lung cancer prevention and contributes to the growing literature on drug‐repurposing strategies in oncology.

## 2 Method

### 2.1 Study Design and Protocol

This systematic review and meta‐analysis of observational studies was conducted in accordance with the Cochrane Collaboration guidelines [25] and reported in compliance with the Preferred Reporting Items for Systematic Reviews and Meta‐Analyses (PRISMA) statement [26]. The study aimed to explore the relationship between Metformin use and the risk of developing lung cancer.

### 2.2 Search Strategy

A systematic search was conducted across multiple databases, including PubMed/MEDLINE, ISI Web of Science, and Scopus, from their inception to January 1, 2026. Additionally, gray literature was identified through a manual search of Google Scholar. The search strategy was developed using a combination of Medical Subject Headings (MeSH) terms and free‐text keywords relevant to the study topic. Search terms were combined using Boolean operators, including: (“Metformin” OR “Biguanides” OR “Hypoglycemic Agents” OR “glucose‐lowering”) AND (“Lung cancer” OR “Lung malignancy” OR “Lung carcinoma” OR “Lung tumor” OR “pulmonary cancer” OR “Lung adenocarcinoma”) AND (“Diabetes” OR “Type 2 diabetes”). The search was applied to the Title and Abstract fields. No language or publication date restrictions were placed to maximize the scope of the search and include studies from diverse settings and periods. Duplicate entries were removed, and relevant studies were screened based on predefined inclusion and exclusion criteria. The detailed search strategy and search lines are provided in the Tables S1 and S2.

### 2.3 Eligibility Criteria

The eligibility criteria for this review were based on the PICO framework: patients with T2DM who used metformin, compared to nonmetformin users, with lung cancer risk as the primary outcome. Only observational studies that investigated the relationship between Metformin use and lung cancer risk in T2DM patients and provided risk estimates such as hazard ratios (HRs), odds ratios (ORs), or relative risks (RRs) with corresponding 95% confidence intervals (CIs) were included. Studies were excluded if they were interventional, experimental (including in vitro, in vivo, or animal models), editorials, commentaries, expert opinions, letters to the editor, review articles, noncohort original studies, or lacked sufficient data for analysis.

### 2.4 Data Extraction

Data extraction was carried out independently by two reviewers (F.H. and K.M.) using a standardized Excel form to maintain consistency and ensure accuracy. The extracted data included study design, sample size, participant characteristics (such as age, sex, and diabetes status), details on Metformin use (including dose and duration), lung cancer risk, and risk estimates (e.g., HRs or ORs) along with their corresponding CIs. In cases where discrepancies arose between the reviewers during data extraction, they were resolved through discussion or consultation with the project supervisor (M.K.). This approach helped ensure the reliability and comprehensiveness of the data included in the analysis.

### 2.5 Quality Assessment

Two independent reviewers (K.M. and F.H.) assessed the quality of the included observational studies using the Newcastle‐Ottawa Scale (NOS). Any discrepancies or disagreements were resolved through discussion or consultation with the project supervisor (M.K.). The NOS version employed a 9‐star rating system to evaluate studies in three main areas: selection, comparability, and outcome assessment. Based on their scores, the studies were classified as high‐quality, fair‐quality, or low‐quality [27].

### 2.6 Statistical Analysis

The RRs and ORs reported by articles were considered equal to HR. A meta‐analysis was performed to estimate the cumulative HR of Metformin therapy for lung cancer, using the random‐effects model (inverse‐variance method). Moreover, the restricted maximum likelihood estimation method was employed to calculate *τ* ^2^. Subgroup analyses were performed based on study design (cohort, nested case–control, and case–control), duration (less than or greater than 3 years), and location (Asia, Europe, and North America). The sensitivity analysis was conducted using leave‐one‐out models to assess the robustness of the synthesized results. The heterogeneity of included studies was evaluated using Cochrane's Q statistic and *I* ^2^ statistic [28], with *I* ^2^ values defined as low (< 25%), moderate (25%–75%), or high (> 75%) heterogeneity. Statistical significance was defined as a two‐sided *p*‐value < 0.05. Visual assessments and statistical Egger [29] and Begg [30] tests were performed to evaluate the risk of publication bias. All statistical analyses were conducted using R Statistical Software's “meta” package (v4.1.2; R Core Team 2021).

## 3 Results

### 3.1 Study Selection

**FIGURE 1:**
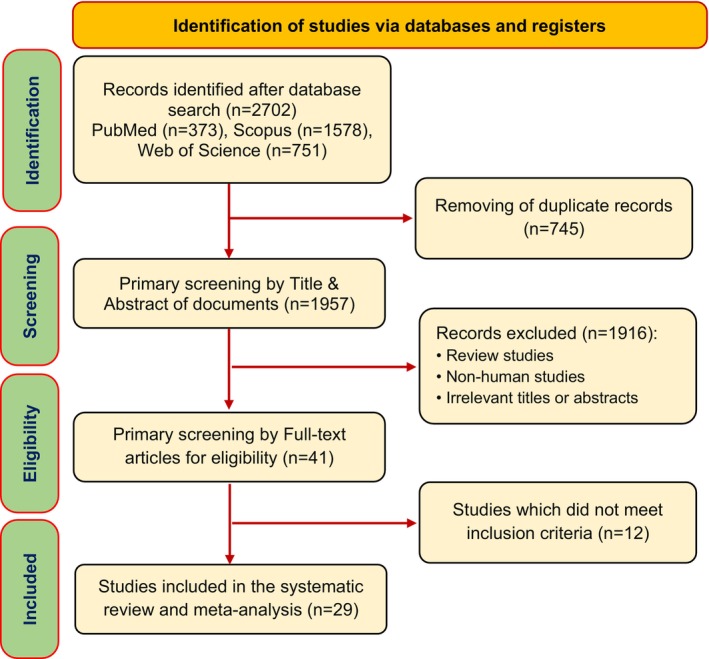
PRISMA flow chart of the study selection process for inclusion studies in the systematic review.

An initial search of four major databases, PubMed (*n* = 373), Web of Science (*n* = 751), and Scopus (*n* = 1578), resulted in 2702 records. After removing 745 duplicates, 1957 unique records were screened by title and abstract. At this stage, 1916 studies were excluded for reasons such as review articles, trials, experimental studies (in vitro or in vivo), letters to the editor, or other irrelevant designs. This left 41 studies for full‐text review to assess eligibility for the meta‐analysis. Of these, 12 were excluded due to insufficient data, incomplete information, or failure to meet the required cohort or case–control design. Ultimately, 29 observational studies [20, 21, 22, 23, 31, 32, 33, 34, 35, 36, 37, 38, 39, 40, 41, 42, 43, 44, 45, 46, 47, 48, 49, 50, 51, 52, 53, 54, 55] met the inclusion criteria and were included in the final meta‐analysis. The PRISMA flow diagram illustrates the study selection process (Figure 1).

### 3.2 Study Characteristics

The review included 29 observational studies: 22 cohort studies [20, 21, 23, 31, 32, 33, 34, 35, 36, 37, 38, 39, 40, 41, 42, 43, 44, 45, 46, 47, 48, 49], two case–control studies [22, 50], and five nested case–control studies [51, 52, 53, 54, 55], published between 2009 and 2024. These studies encompassed a broad range of participants (*n* = 433 to 342 045), with ages ranging from 18 to 90 years, although most populations were aged 40 years or older. Several studies reported detailed age distributions, including mean ages or categorical age groups. Both male and female participants were represented. Effect estimates were reported as HRs, ORs, or RRs, each with corresponding 95% CIs. Overall, findings were mixed; however, the majority of studies suggested a protective association. Specifically, 22 studies (75.8%) reported a statistically significant reduction in lung cancer risk associated with metformin use, with effect estimates ranging from 0.29 to 0.89. In contrast, seven studies (24.1%) found no statistically significant association, as their confidence intervals included the null value (1.0).

Most studies adjusted for a wide range of potential confounders, including age, sex, smoking status, alcohol consumption, body mass index (BMI), duration of diabetes, HbA1c levels, comorbid conditions (such as chronic obstructive pulmonary disease and hypertension), socioeconomic status, and concomitant medication use. The inclusion of these variables reflects substantial efforts to minimize confounding and strengthen the validity of the observed associations between metformin use and lung cancer risk (Table 1).

**TABLE 1:** Basic characteristics of included cohort studies in the meta‐analysis.

Author/year	Country	Study type	Sample size	Study duration (m)	Age (years)	Gender	Effect size (95% CI)	Adjustment
Sakoda et al. [20]	USA	Cohort—retrospective	21 526	NR	59.3 ± 10.1 (> 40)	Male: 52.3% Female: 47.7%	HR: 1.02 (0.85–1.22)	Race/ethnicity, smoking status, alcohol, income, education level, DM duration, BMI, HbA1c, creatinine
Kang et al. [31]	Korea	Cohorts	25 791	NR	40–79	Male: 52% Female: 48%	HR: 0.89 (0.81–0.98)	Age, sex, income, BMI, smoking, alcohol, medication use, CCI
Kim et al. [32]	Korea	Cohorts	4889	≥ 3	40–69	Male (100%)	HR: 1.287 (0.979–1.691)	Sex, age, BMI, smoking, alcohol, HTN, TC, physical activity, ALT
Kim et al. [32]	Korea	Cohorts	3917	≥ 3	40–69	Female (100%)	HR: 0.664 (0.374–1.177)	Sex, age, BMI, smoking, alcohol, HTN, TC, physical activity, ALT
Smiechowski et al. [51]	UK	Nested case–control	6611	NR	> 40	Male: 55% Female: 45%	RR: 0.94 (0.76–1.17)	DM duration, HbA1c, obesity, smoking, alcohol, previous cancer, COPD, asthma, drugs
Vicentini et al. [21]	Italy	Cohorts	2875	≥ 36	20–84	Male: 56% Female: 44%	IRR: 1.14 (0.71–1.85)	Age, sex, citizenship
Tsai et al. [33]	Taiwan	Cohorts	22 153	> 1	NR	Male: 53% Female: 47%	HR: 0.693 (0.588–0.812)	Age, sex, modified CCI score
Lai et al. [34]	Taiwan	Cohorts	16 616	NR	> 20	Male: 56% Female: 44%	HR: 0.43 (0.29–0.63)	Age, sex, comorbidities (obesity, pulmonary tuberculosis, COPD, pneumoconiosis, asbestosis, tobacco use)
Ruiter et al. [35]	Netherlands	Cohorts	85 289	NR	NR	Male: 54% Female: 46%	HR: 0.87 (0.84–0.91)	Age, sex, drugs, hospitalizations period in the year before the start of OGLD
Yang et al. [36]	Hong Kong	Cohorts	1266	92.7	45–67	NR	HR: 0.46 (0.28–0.74)	Age, sex, employment status, smoking status, alcohol intake, DM duration, BMI ($27.6 and 24.0 kg/m^2^), systolic BP, HbA1c
Yang et al. [36]	Hong Kong	Cohorts	433	92.7	45–67	NR	HR: 0.29 (0.13–0.61)	Age, sex, employment status, smoking status, alcohol intake, DM duration, BMI, systolic BP, HbA1c
Tsilidis et al. [37]	UK	Cohorts	51 484	> 12	35–90	Male: 56% Female: 44%	HR: 0.85 (0.68–1.07)	Age, sex, smoking, BMI, alcohol, use of aspirin, NSAIDs, statins, DM duration
Libby et al. [23]	Scotland	Cohorts	4085	NR	35–55 (24.5%) 56–63 (23.6%) 64–69 (21.2%) 70–76 (19.1%) 77–10 (11.6%)	Male: 54.1% Female: 45.9%	HR: 0.46 (0.40–0.53)	Sex, age, BMI, HbA1c, smoking, drug use
Hsieh et al. [38]	Taiwan	Cohorts	3963	NR	NR	Male: 51% Female: 48.9%	OR: 1.570 (1.11–2.22)	Age, sex
Bodmer et al. [22]	UK	Case–control	3312	> 40	NR	Male: 58% Female: 42%	OR: 1.21 (0.97–1.50)	Age, sex, smoking status, alcohol intake, BMI, comorbidities (CHF, IHD, HTN, DM, COPD, stroke, dyslipidemia)
Kowall et al. [39]	Germany	Cohorts	55 988	NR	30–89	Male: 55% Female: 45%	HR: 0.68 (0.46–0.99)	Age, sex, country, time between first diagnosis of DM and prescription of first DM medications, obesity, HTN, hyperlipidemia, complications, use of antithrombotics, aspirin, statin, NSAIDS, OCP
Tseng et al. [40]	Taiwan	Cohorts	15 414	NR	NR	Male: 57% Female: 43%	HR: 0.717 (0.584–0.881)	Age, sex, smoking status, alcohol intake, comorbidities, medications
Farmer et al. [41]	UK	Cohorts	6105	NR	30–90	Male: 59% Female: 41%	HR: 0.99 (0.71–1.37)	Age, sex, smoking status, alcohol intake, HBA1C, BMI, comorbidities, medications
Ferrara et al. [42]	USA	Cohorts	109 802	NR	> 40	NR	HR: 1.0 (0.8–1.1)	Age, DM medications, year of cohort entry, sex, race/ethnicity, income, current smoking, HbA1c, DM duration, new DM diagnosis, creatinine, HF
Luo et al. [43]	USA	Cohorts	926	NR	50–79	Female (100%)	HR: 1.32 (0.76–2.28)	Age, ethnicity, education, smoking status, BMI, physical activity, alcohol, waist‐to‐hip ratio, diet, hormone therapy.
Wang et al. [55]	Taiwan	Nested case–control	10 767	NR	NR	NR	HR: 1.11 (0.94–1.47)	Age, sex, and occupation
Murff et al. [44]	USA	Cohorts	42 217	NA	NA	Male: 97% Female: 3%	HR: 0.87 (0.79–0.96)	HbA1c, anti‐hypertensive and lipid‐lowering medications, BMI
Oh et al. [54]	South Korea	Nested case–control	39 092	> 90 days	> 18	Male: 53% Female: 47%	HR: 1.23 (0.94–1.39)	Propensity score (PS) matching
Dankner et al. [45]	Israel	Cohorts	313 460	NR	21–87	Male: 47% Female: 53%	HR: 1.02 (0.59–1.78)	Treatments with other glucose‐lowering medications, age, sex, ethnicity, socioeconomic status, smoking
Sung et al. [46]	China	Cohorts	34 095	> 6 months	> 18	Male: 46.5% Female: 53.5%	HR: 0.63 (0.51–0.78)	Age, sex, comorbidities, medications
Dickerman et al. [47]	UK	Cohorts	9835	NR	> 30	Male: 54% Female: 46%	HR: 0.75 (0.49–1.17)	Age, sex, BMI, smoking, HbA1c, cerebrovascular disease, CVD, medications
Wang et al. [50]	Taiwan	Case–control	342 045	> 2	> 20	Male: 54% Female: 46%	HR: 0.977 (0.951–1.005)	Age, sex, comorbidities
Staa et al. [52]	Netherlands	Nested case–control	109 708	NR	> 40	Male: 56.3% Female: 43.7%	HR: 1.06 (0.80–1.41)	Age, sex, calendar year
Jonusas et al. [48]	Lithuania	Cohorts	26 778	NR	> 40	Male: 34.4% Female: 65.6%	HR: 0.69 (0.55–0.86)	Age and sex
Tabernacki et al. [49]	USA	Cohorts	250 615	NR	NR	Male: 47.2% Female: 52.8%	HR: 0.764 (0.725–0.806)	Demographics, BMI, HbA1c, preexisting medical conditions, family and personal history of cancers and lung nodules, lifestyle factors (e.g., smoking and alcohol use), environmental exposures
Zhou et al. [53]	China	Nested case–control	27 209	NR	NR	NR	OR: 0.988 (0.914–1.069)	NR

Abbreviations: ALT, alanine transaminase; BMI, body mass index; BP, blood pressure; CCI, Charlson comorbidity index; CI, confidence interval; COPD, chronic obstructive pulmonary disease; CVD, cardiovascular diseases; DM, diabetes mellitus; HbA1c, hemoglobin A1c; HF, heart failure; HR, hazard ratio; HTN, hypertension; IRR, incidence rate ratio; NR, nonreported; NSAIDs, nonsteroidal anti‐inflammatory drugs; OR, odds ratio; PS, propensity Score; RR, risk ratio; TC, total cholesterol.

### 3.3 Quality Assessments

The quality assessment of the included cohort and case–control studies, as outlined in Tables 2 and 3, indicates a rigorous evaluation based on predefined criteria, including cohort representativeness, exposure ascertainment, comparability, follow‐up adequacy, and response rates. Most studies scored highly, achieving ratings of 7 or 8 out of 10, demonstrating strong methodological integrity. However, a few studies received lower scores (e.g., 4 to 6), mainly due to limitations in case definition, exposure ascertainment, or follow‐up duration. These variations underscore differences in study design and execution, which should be considered when interpreting the overall findings and conclusions.

**TABLE 2:** Quality assessment of the included cohort studies.

Cohort studies	Q1	Q2	Q3	Q4	Q5	Q6	Q7	Q8	Overall
Sakoda et al. [20]	*	*	*	*	*	*	*	*	8
Kang et al. [31]	*	*	*	*	*	*	—	*	7
Kim et al. [32]	*	*	*	*	*	*	*	*	8
Vicentini et al. [21]	*	*	*	*	*	*	—	*	7
Tsai et al. [33]	*	*	*	*	*	*	*	*	8
Lai et al. [34]	*	*	*	*	*	*	*	*	8
Ruiter et al. [35]	*	*	*	*	*	*	*	*	8
Yang et al. [36]	*	*	*	*	*	*	—	*	7
Tsilidis et al. [37]	*	*	*	*	*	*	*	*	8
Libby et al. [23]	*	*	*	*	*	*	*	*	8
Hsieh et al. [38]	*	*	*	*	*	*	—	—	6
Kowall et al. [39]	*	*	*	*	*	*	*	*	8
Tseng et al. [40]	*	*	*	*	*	*	—	*	7
Farmer et al. [41]	*	*	*	*	*	*	—	*	7
Ferrara et al. [42]	*	*	*	*	*	*	—	*	7
Luo et al. [43]	*	*	*	*	*	*	—	*	7
Murff et al. [44]	*	*	*	*	*	*	—	*	7
Dankner et al. [45]	*	*	*	*	*	*	—	*	7
Sung et al. [46]	*	*	*	*	*	*	*	*	8
Dickerman et al. [47]	*	*	*	*	*	*	—	*	7
Jonusas et al. [48]	*	*	*	*	*	*	*	*	8
Tabernacki et al. [49]	*	*	*	*	*	*	*	*	8

*Note:* Q1: Representativeness of the exposed cohort. Q2: Selection of the nonexposed cohort. Q3: Ascertainment of exposure. Q4: Demonstration that outcome of interest was not present at the start of the study. Q5: Comparability of cohorts on the basis of the design or analysis. Q6: Assessment of outcome. Q7: Was follow‐up long enough for outcomes to occur (> 10 years). Q8: Adequacy of follow‐up of cohorts (loss‐to‐follow‐up < 20%). An asterisk (*) indicates that the study meets the Newcastle–Ottawa Scale criterion.

**TABLE 3:** Quality assessment of the included Case–Control studies.

Case–control studies	Q1	Q2	Q3	Q4	Q5	Q6	Q7	Q8	Overall
Smiechowski et al. [51]	*	*	*	*	*	*	*	*	8
Bodmer et al. [22]	*	*	*	*	*	*	*	—	7
Wang et al. [55]	*	—	—	—	*	*	*	*	5
Oh et al. [54]	*	*	*	—	*	*	*	*	7
Wang et al. [50]	*	*	*	*	*	*	*	*	8
Staa et al. [52]	*	*	*	*	*	*	*	—	7
Zhou et al. [53]	*	*	*	—	—	—	*	—	4

*Note:* Q1: Is the case definition adequate? Q2: Representativeness of the cases. Q3: Selection of controls. Q4: Definition of controls. Q5: Comparability of cases and controls based on the design or analysis. Q6: Ascertainment of exposure. Q7: Same method of ascertainment for cases and controls. Q8: Nonresponse rate. An asterisk (*) indicates that the study meets the Newcastle–Ottawa Scale criterion.

### 3.4 Meta‐Analysis

**FIGURE 2:**
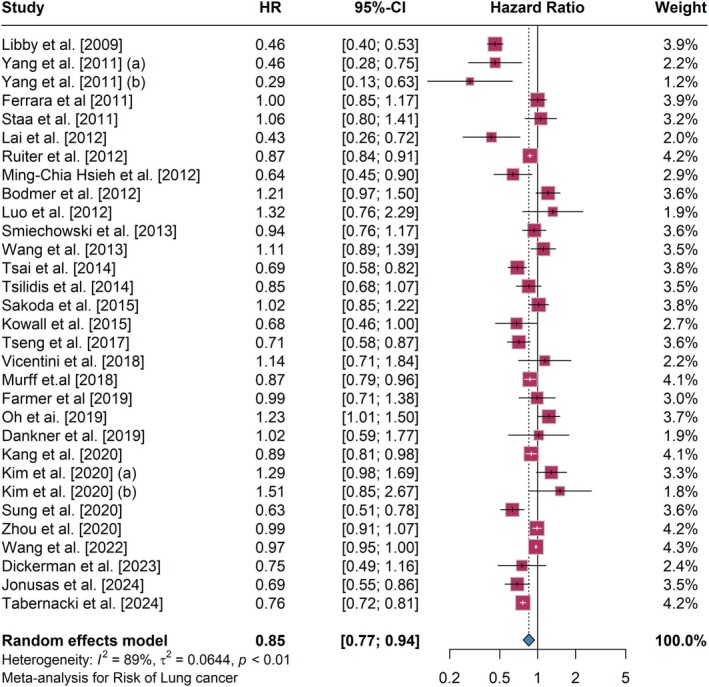
Forrest plot demonstrating the overall effects of hazard ratio (HR) with 95% confidence intervals (CI) for the association between Metformin users compared with nonusers and the risk of lung cancer in diabetic individuals.

Meta‐analysis of 29 studies using a random‐effects model showed a significantly reduced risk of lung cancer among the cases who used Metformin compared to non‐users (HR: 0.85; 95% CI: [0.77, 0.94]; *p* = 0.002), while high between‐study heterogeneity was observed (*I* ^2^ = 88.7%) (Figure 2).

**FIGURE 3:**
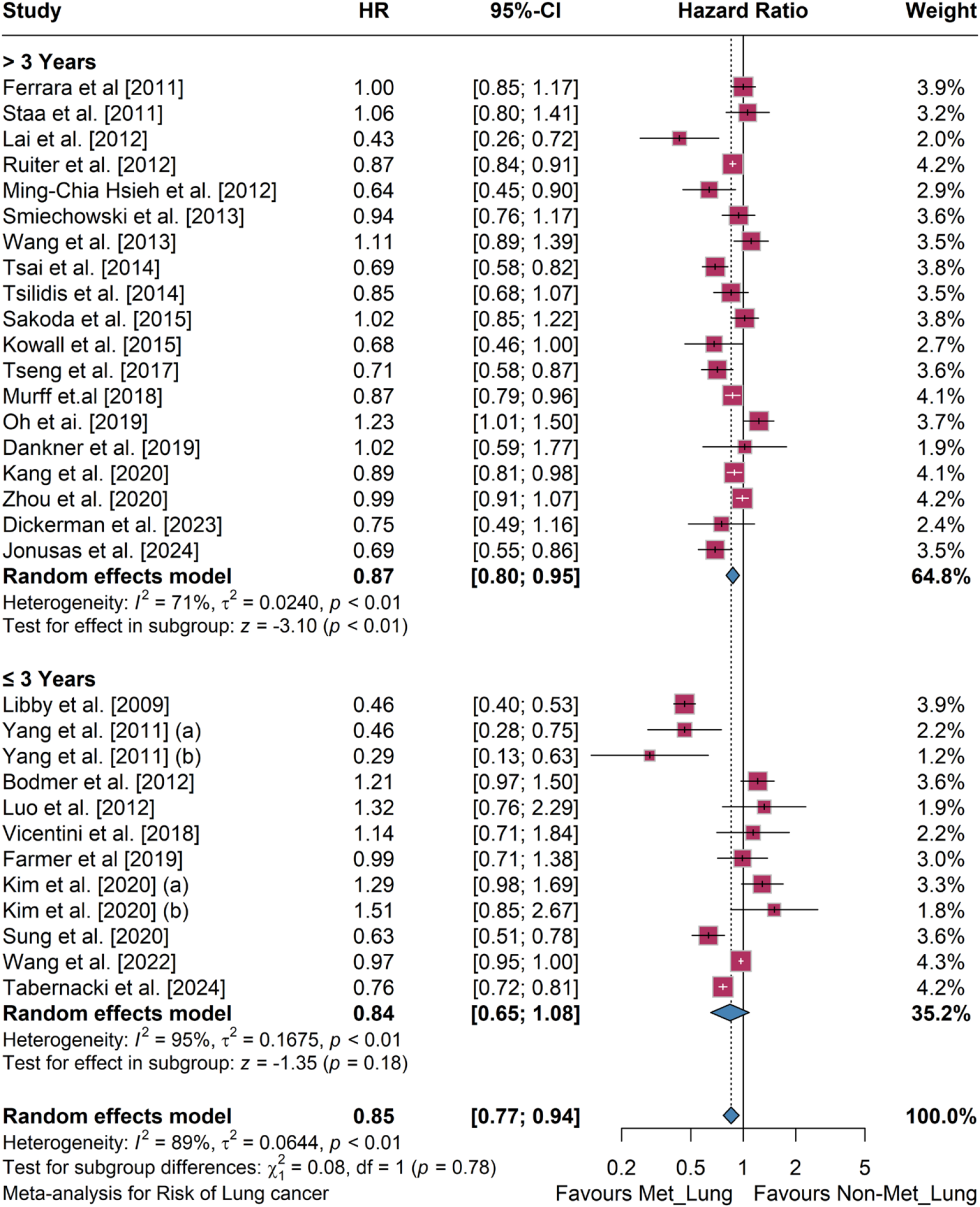
Forrest plot demonstrating the hazard ratio (HR) with 95% confidence intervals (CI) based on follow‐up duration for the association between Metformin users compared with nonusers and the risk of lung cancer in diabetic individuals.

Based on study duration, design, and geographical location, subgroup analyses were performed to assess the association between Metformin use and lung cancer risk. Analysis of 19 studies with a duration of more than 3 years revealed a significant reduction in lung cancer risk among Metformin users compared to nonusers (HR: 0.87, 95% CI: [0.89–0.95]; *p* < 0.01, *I* ^2^ = 75%), whereas no significant association was found in studies with durations shorter than 3 years (Figure 3).

**FIGURE 4:**
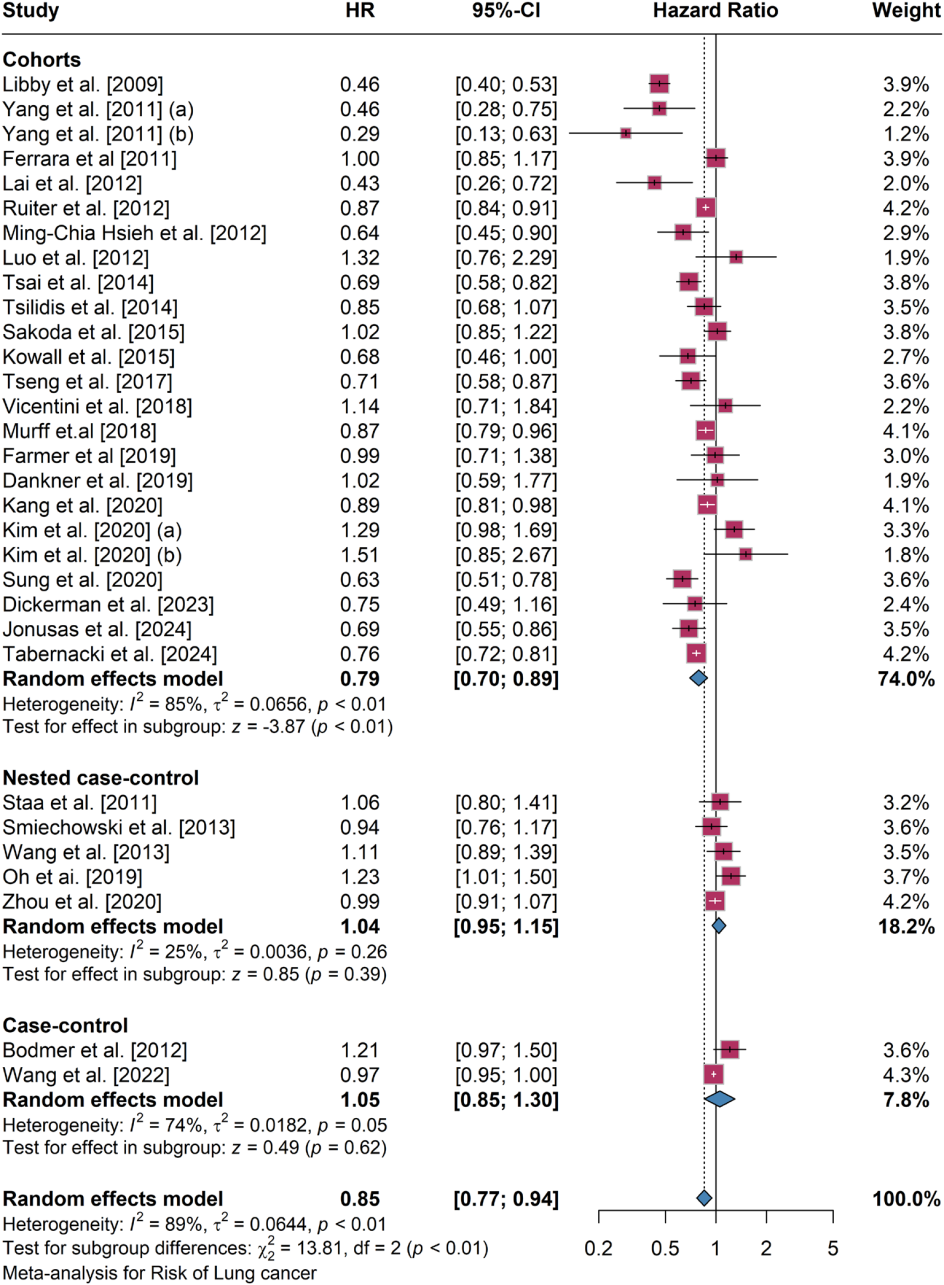
Forrest plot demonstrating the hazard ratio (HR) with 95% confidence intervals (CI) based on study design for the association between Metformin users compared with nonusers and the risk of lung cancer in diabetic individuals.

Regarding study design, cohort studies showed a significant protective effect of Metformin against lung cancer (HR: 0.79, 95% CI: [0.70–0.89]; *p* < 0.01), whereas nested case–control and case–control studies did not report significant associations (Figure 4).

**FIGURE 5:**
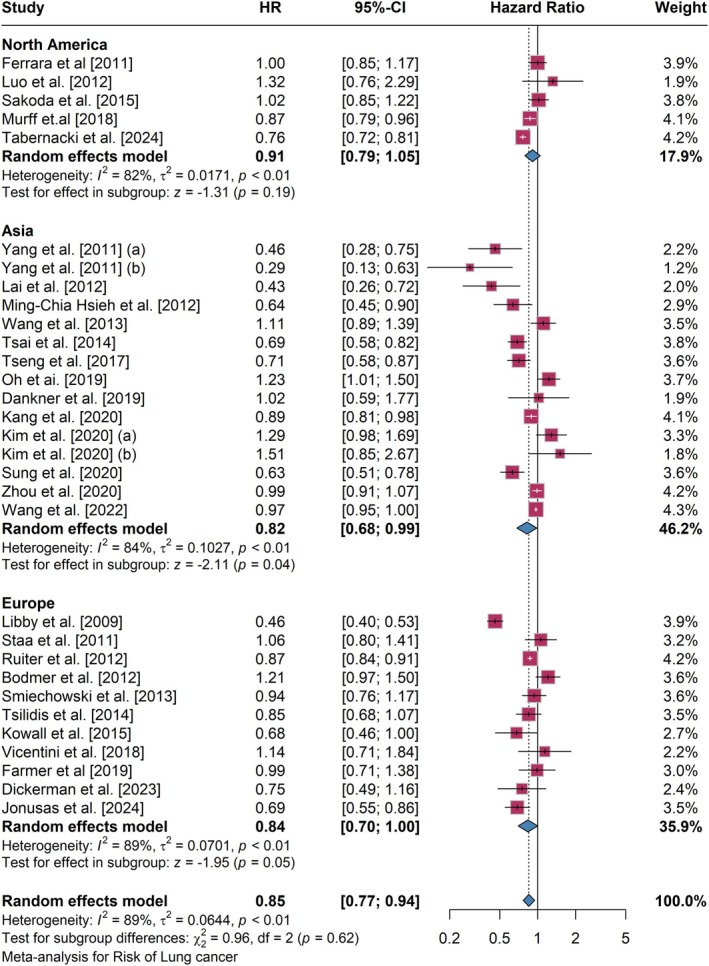
Forrest plot demonstrating the hazard ratio (HR) with 95% confidence intervals (CI) based on study location (continents) for the association between Metformin users compared with non‐users and the risk of lung cancer in diabetic individuals.

Geographical subgroup analyses revealed a significantly lower risk of lung cancer among Metformin users in studies conducted in Asia (HR: 0.82; 95% CI: 0.68–0.98; *p* = 0.04). A similar trend was observed in European studies (HR: 0.84; 95% CI: 0.70–1.00; *p* = 0.05), indicating a borderline statistically significant association. In contrast, studies conducted in North America did not demonstrate a significant association between Metformin use and lung cancer risk (Figure 5).

Heterogeneity remained high across most subgroups (*p* < 0.05), except for nested case–control studies, where heterogeneity was relatively low (*I* ^2^ = 24.9%). Detailed findings of this meta‐analysis of the risk of lung cancer in Metformin users are presented in Table 4.

**TABLE 4:** Findings of meta‐analysis of the risk of lung cancer in metformin users.

Group	Number of ES	Pooled HRs	95% CI	*p* ^a^	Heterogeneity (*I* ^2^)	*p* ^b^
Overall	31	0.85	[0.77–0.94]	0.002*	88.7%	< 0.001
Study designs						
Cohorts	24	0.79	[0.70–0.89]	< 0.001*	85.0%	< 0.01
Nested case–control	5	1.04	[0.95–1.15]	0.39	24.9%	0.26
Cass–control	2	1.05	[0.85–1.30]	0.62	74.3%	0.05
Follow‐up duration						
> 3 years	19	0.87	[0.80–0.95]	< 0.001*	70.5%	< 0.01
≤ 3 years	12	0.84	[0.65–1.08]	0.18	94.6%	< 0.01
Study location						
Asia	15	0.82	[0.68–0.99]	0.04*	82.4%	< 0.01
Europe	11	0.84	[0.70–1.00]	0.05	84.3%	< 0.01
North America	5	0.91	[0.79–1.05]	0.19	89.4%	< 0.01

*Note:* Bold values with an asterisk (*) indicate statistically significant (*p* < 0.05).

Abbreviations: CI, confidence interval; ES, effect size.

^a^ *p*‐value of Hazard ratios based on the random effects model.

^b^ *p*‐value of heterogeneity based on Cochran's Q.

### 3.5 Sensitivity Analysis

**FIGURE 6:**
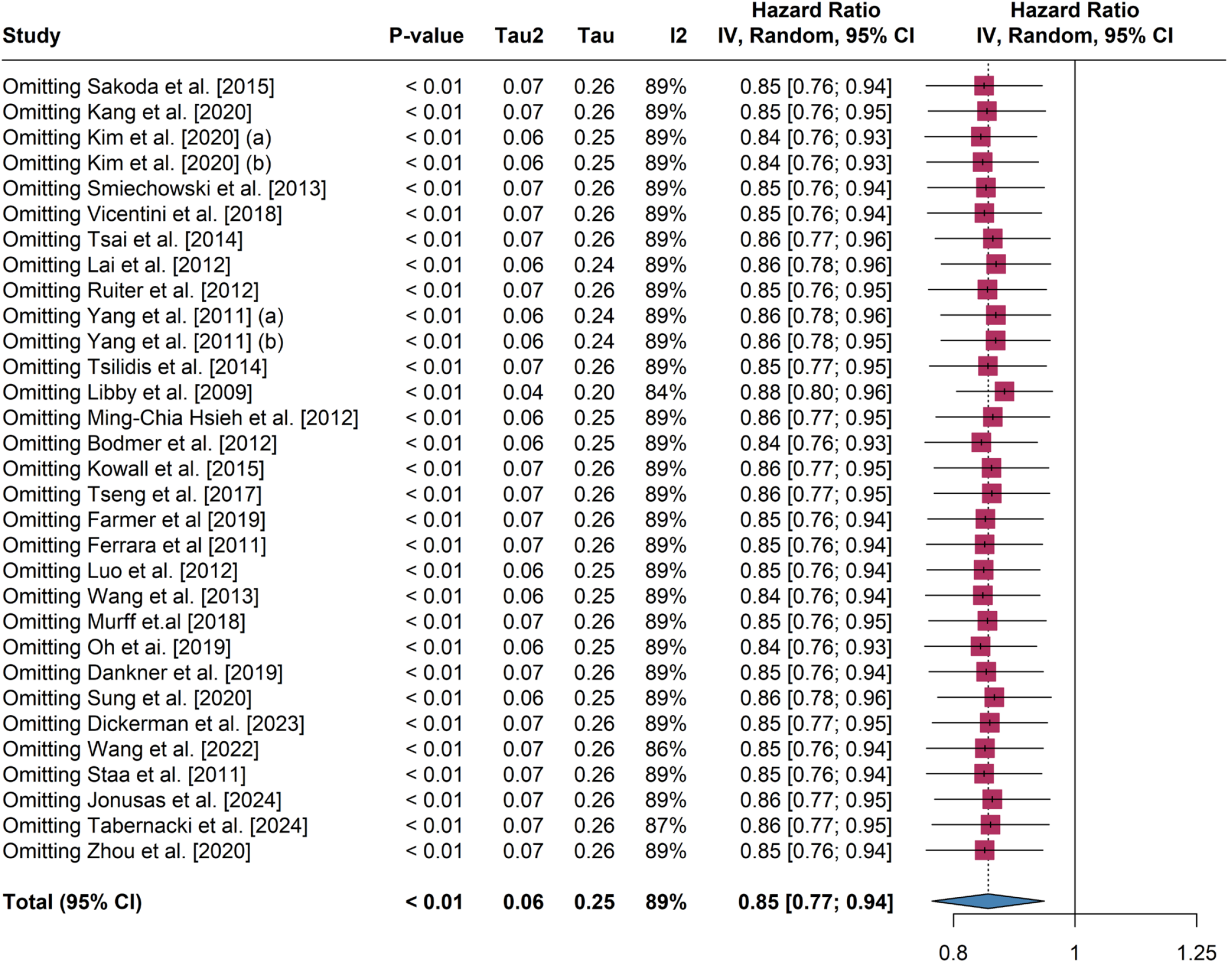
Forest plot of the sensitivity analysis based on the leave‐one‐out.

The sensitivity analysis using the leave‐one‐out method was performed to assess the robustness of the findings. Removing any of the included articles did not change the significance of the pooled estimate, which indicates the robustness of the current review result (Figure 6).

### 3.6 Risk of Publication Bias

**FIGURE 7:**
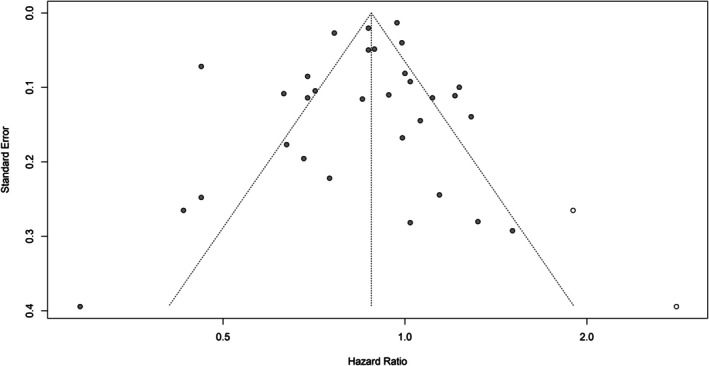
Funnel plot of the risk of publication bias of included articles in the meta‐analysis.

The risk of publication bias was evaluated through visual inspection and statistical tests. Figure 7 shows the funnel plot of the included studies. The funnel plot appears symmetrical, indicating no publication bias, as confirmed by the Egger (*p* = 0.223) and Begg (*p* = 0.798) tests.

### 3.7 Meta‐Regression

To address the high between‐study heterogeneity, we conducted a meta‐regression using the year of publication and study duration as covariates (Table S3). However, none of the above variables were significantly responsible for the observed heterogeneity.

## 4 Discussion

The current systematic review and meta‐analysis aimed to investigate the possible association between Metformin and the risk of lung cancer in patients with T2DM. The pooled analysis of 29 observational studies demonstrated a significant association: Metformin use was linked to a 15% reduction in lung cancer risk compared with non‐use, suggesting a potential protective effect. However, the high heterogeneity among studies (*I* ^2^ = 88.7%) necessitates cautious interpretation. This variability likely reflects differences in study design, population demographics, Metformin dosage and duration, and confounding factors such as smoking and comorbidities.

The subgroup analyses provide important insights into the association between Metformin use and lung cancer risk. Significant protective effects were observed in cohort studies, whereas no significant associations were detected in case–control or nested case–control studies. This discrepancy may be attributable to differences in study design, as cohort studies are generally less susceptible to recall and selection biases and may better establish temporal relationships between Metformin exposure and lung cancer outcomes. Geographically, studies conducted in Asia demonstrated a significant reduction in lung cancer risk among Metformin users, while studies from Europe showed a borderline statistically significant association, and no significant effect was observed in North American studies. Several factors may contribute to these regional differences, including variations in genetic susceptibility, environmental exposures, healthcare systems, prescribing patterns, and lifestyle factors. For example, exposure to established lung cancer risk factors such as air pollution, arsenic contamination, occupational carcinogens, and smoking behaviors differs substantially across regions and may modify the potential effects of Metformin. Furthermore, ethnic and genetic differences affecting metabolic pathways, insulin resistance, and drug response could influence the magnitude of Metformin's anticancer effects [56]. Duration of exposure may also be important, as studies with follow‐up periods exceeding 3 years showed significant risk reductions, suggesting that prolonged Metformin use may be necessary to exert measurable anticancer benefits. Nevertheless, the substantial heterogeneity observed across most subgroup analyses indicates that additional unmeasured confounders and methodological differences likely contribute to the variability in findings. Future well‐designed prospective studies with detailed information on genetic background, environmental exposures, and long‐term Metformin use are needed to clarify these sources of heterogeneity and better define the populations most likely to benefit from Metformin's potential protective effects against lung cancer.

As noted in our studies, the role of Metformin in lung cancer prevention remains a topic of significant interest, with mixed evidence across multiple meta‐analyses. Yao et al. [17] observed a reduced lung cancer risk with Metformin use in diabetic patients, particularly in European populations and cohort studies that adjusted for smoking, though notable heterogeneity was present. Similarly, Wang et al. [18] found a strong protective effect, with Metformin users exhibiting a 45% lower odds of lung cancer compared to nonusers. Zhang et al. [19] corroborated these findings, linking Metformin therapy to reduced risks of lung and respiratory cancers, albeit with moderate variability after adjustment for smoking. However, Sakoda et al. [20] reported no overall association, though their findings highlighted potential protective effects among never‐smokers and long‐term users, suggesting a complex interplay between metformin, smoking, and cancer histology. Collectively, these studies underscore the promising yet nuanced relationship between Metformin and lung cancer risk, emphasizing the need for well‐designed randomized controlled trials to validate and clarify these observational findings.

The potential role of Metformin in reducing lung cancer risk, as observed in these studies, is thought to be linked to its cellular and molecular mechanisms of action. Metformin activates AMP‐activated protein kinase (AMPK), a key regulator of cellular energy homeostasis, and inhibits the mammalian target of rapamycin (mTOR) pathway, which promotes cell growth and proliferation [57, 58, 59]. By suppressing mTOR signaling, Metformin may inhibit cancer cell growth and induce apoptosis [60]. Furthermore, Metformin has been shown to reduce survivin, an anti‐apoptotic protein frequently overexpressed in malignant cells, via proteasomal degradation [61]. Metformin has been found to trigger ferroptosis in lung cancer cells via activating the Nrf2/HO‐1 signaling pathway [62]. Additionally, Metformin improves insulin sensitivity and lowers circulating insulin levels, reducing the pro‐growth effects of insulin and insulin‐like growth factors (IGFs) on cancer cells [63, 64]. It also modulates inflammation and oxidative stress [65, 66], both of which are implicated in cancer development and may exert indirect anti‐tumor effects by altering the tumor microenvironment [67]. The combined biological mechanisms offer a compelling basis for the protective effects observed in some studies, suggesting that Metformin may reduce lung cancer risk and progression in diabetic patients and highlighting its potential as an effective protective medication for this population [68, 69]. However, variability in study results highlights the need for further research to confirm its protective role and clarify the influence of factors like study design, patient demographics, and cancer subtypes.

### 4.1 Strengths, Limitations, and Future Suggestions

The strength of this study lies in its large sample size and the inclusion of diverse observational studies, which provide a comprehensive analysis of metformin's potential effect on lung cancer risk. Additionally, subgroup analyses by study design, duration, and geographic location provide valuable insights into factors that may influence the association between metformin use and lung cancer. One limitation of this study is the high heterogeneity among the included studies, which may reflect differences in study design, population characteristics, and metformin use. Furthermore, age‐ and sex‐specific subgroup analyses could not be conducted due to insufficiently detailed or inconsistently reported data across the included studies, limiting the ability to assess whether the association varies by these important demographic factors. Additionally, the observational nature of the included studies limits the ability to establish causality and fully control for potential confounding factors such as smoking and comorbidities.

Future investigations should focus on stratified analyses by age and sex, account for potential confounders, and explore the dose–response relationship between metformin and lung cancer risk. These steps will help establish whether the observed association reflects a causal relationship or is influenced by external factors.

## 5 Conclusion

This systematic review and meta‐analysis suggest that Metformin use is associated with a 15% reduction in the risk of lung cancer in patients with T2DM. The pooled results indicate a potential protective effect, particularly with long‐term Metformin use in specific populations, such as those in Asia. However, the high heterogeneity among studies and the observational nature of the evidence highlight the need for caution in interpreting these findings. Confounding factors such as smoking and comorbidities may influence the observed association. Future research, particularly well‐designed randomized controlled trials and stratified analyses, is essential to confirm these results, establish causality, and explore the underlying mechanisms of metformin's protective role in lung cancer prevention.

## Author Contributions

**Mehdi Karimi:** conceptualization, investigation, funding acquisition, writing – original draft, writing – review and editing, validation, methodology, visualization, software, formal analysis, project administration, supervision, resources, data curation. **Kiana Mohammadian:** writing – original draft, writing – review and editing, conceptualization, investigation, validation, project administration, funding acquisition, methodology. **Seyed Morteza Ali Pourfaraji:** writing – original draft, formal analysis, software, data curation, supervision, resources, project administration, investigation, validation, methodology, visualization, funding acquisition. **Masoud Mortezazadeh:** writing – review and editing, conceptualization, investigation, validation, supervision, project administration, methodology, funding acquisition, software. **Farnaz Hooshmand:** writing – original draft, funding acquisition, investigation, conceptualization, writing – review and editing, supervision, project administration.

## Funding

The authors have nothing to report.

## Ethics Statement

The authors have nothing to report.

## Consent

The authors have nothing to report.

## Conflicts of Interest

The authors declare no conflicts of interest.

## Supporting information


**Table S1:** Search and query words in databases.
**Table S2:** Search line in the databases.
**Table S3:** The findings of meta‐regression analyses (Lung cancer).

## Data Availability

All data used in this meta‐analysis were extracted from published studies. The datasets supporting the findings of this study are available from the original sources cited in the manuscript. Additional information can be provided by the corresponding author upon reasonable request.
