# Supplementary material for: Association Between Metformin Use and Risk of Lung Cancer in Patients With Type 2 Diabetes: A Systematic Review and Meta‐Analysis
Source: Cancer Rep (Hoboken). 2026 Aug 11;9(8):e70651. doi: 10.1002/cnr2.70651 (PMC13461527; doi:10.1002/cnr2.70651)
Supplement: Supplementary file 1 [file CNR2-9-e70651-s001.docx]

**Supplementary Materials**

**Title:**

**Association between Metformin Use and Risk of Lung Cancer in Patients with Type 2 Diabetes: A Systematic Review and Meta-Analysis**

**Supplementary Table 1.** Search and query words in databases

| **Databases** | **Search and query words** |
| --- | --- |
| **Metformin** | “Metformin” OR “biguanides” OR “hypoglycemic agents” |
| **Lung cancer** | "Lung cancer" OR “lung malignancy” OR “lung carcinoma” OR “lung tumor” OR “pulmonary cancer” OR “carcinoma of lung” |
| **Full Search Strategy** | “Metformin” OR “Biguanides” OR “Hypoglycemic Agents” **AND** "Lung cancer" OR "Lung malignancy" OR "Lung carcinoma" OR "Lung tumor" OR "pulmonary cancer" OR “Carcinoma of Lung” |

**Supplementary Table 2.** Search line in the databases.

| **Databases** | **Search line** | **No. of Records** |
| --- | --- | --- |
| **PubMed** | ("Metformin"[Title/Abstract] OR "Biguanides"[Title/Abstract] OR "Hypoglycemic Agents"[Title/Abstract] [Title/Abstract]) AND ("Lung cancer"[Title/Abstract] OR "Lung malignancy"[Title/Abstract] OR "Lung carcinoma"[Title/Abstract] OR "Lung tumor"[Title/Abstract] OR "pulmonary cancer"[Title/Abstract] OR "Carcinoma of Lung"[Title/Abstract]) | 373 |
| **ISI WoS** | Results for “Metformin” OR “Biguanides” OR “Hypoglycemic Agents” (Topic) AND "Lung cancer" OR "Lung malignancy" OR "Lung carcinoma" OR "Lung tumor" OR "pulmonary cancer" OR “Carcinoma of Lung” (Topic) | 751 |
| **Scopus** | (TITLE-ABS-KEY ("Metformin" OR "Biguanides" OR "Hypoglycemic Agents") AND TITLE-ABS-KEY ("Lung cancer" OR "Lung malignancy" OR "Lung carcinoma" OR "Lung tumor" OR "pulmonary cancer" OR "Carcinoma of Lung")) | 1578 |

**Supplementary Table 3.** The findings of meta-regression analyses (Lung cancer)

| **Predictor variable** | **Amount of heterogeneity accounted for** | **Residual heterogeneity  (I^2^)** | **Test of the moderator (P-value)** |
| --- | --- | --- | --- |
| Year of publication | 0.0 % | 94.0 % | 0.21 |
| Follow-up time | 0.0 % | 93.58 % | 0.52 |
